# A non-randomised feasibility study of an intervention to optimise medicines at transitions of care for patients with heart failure

**DOI:** 10.1186/s40814-021-00819-x

**Published:** 2021-03-26

**Authors:** Beth Fylan, Hanif Ismail, Suzanne Hartley, Chris P. Gale, Amanda J. Farrin, Peter Gardner, Jonathan Silcock, David P. Alldred, on behalf of the ISCOMAT Programme Team

**Affiliations:** 1 School of Pharmacy and Medical Sciences, University of Bradford, Richmond Road, Bradford, BD7 1DP UK; 2 NIHR Yorkshire and Humber Patient Safety Translational Research Centre. Bradford Institute for Health Research, Temple Bank House, Bradford, BD9 6RJ UK; 3 Wolfson Centre for Applied Health Research, Bradford, BD9 6RJ UK; 4 Clinical Trials Research Unit, Leeds Institute of Clinical Trials Research, University of Leeds, Leeds, LS2 9JT UK; 5 Leeds Institute of Cardiovascular and Metabolic Medicine, University of Leeds, Leeds, LS2 9JT UK; 6 School of Healthcare, University of Leeds, Leeds, LS2 9JT UK

**Keywords:** Heart failure, Cardiology, Care transitions, Complex intervention, Clinical trials, Feasibility studies

## Abstract

**Background:**

Heart failure affects 26 million people globally, and the optimal management of medicines is crucial for patients, particularly when their care is transferred between hospital and the community. Optimising clinical outcomes requires well-calibrated cross-organisational processes with staff and patients responding and adapting to medicines changes. The aim of this study was to assess the feasibility of implementing a complex intervention (the Medicines at Transitions Intervention; MaTI) co-designed by patients and healthcare staff. The purpose of the intervention was to optimise medicines management across the gaps between secondary and primary care when hospitals handover care. The study objectives were to (1) assess feasibility through meeting specified progression criteria to proceed to the trial, (2) assess if the intervention was acceptable to staff and patients, and (3) determine whether amendment or refinement would be needed to enhance the MaTI.

**Methods:**

The feasibility of the MaTI was tested in three healthcare areas in the North of England between July and October 2017. Feasibility was measured and assessed through four agreed progression to trial criteria: (1) patient recruitment, (2) patient receipt of a medicines toolkit, (3) transfer of discharge information to community pharmacy, and (4) offer of a community pharmacy medicines review/discussion or medicines reconciliation. From the cardiology wards at each of the three NHS Acute Trusts (sites), 10 patients (aged ≥ 18 years) were recruited and introduced to the ‘My Medicines Toolkit’ (MMT). Patients were asked to identify their usual community pharmacy or nominate a pharmacy. Discharge information was transferred to the community pharmacy; pharmacists were asked to reconcile medicines and invited patients for a medicines use review (MUR) or discussion. At 1 month following discharge, all patients were sent three questionnaire sets: quality-of-life, healthcare utilisation, and a patient experience survey. In a purposive sample, 20 patients were invited to participate in a semi-structured interview about their experiences of the MaTI. Staff from hospital and primary care settings involved in patients’ care were invited to participate in a semi-structured interview. Patient and staff interviews were analysed using Framework Analysis. Questionnaire completion rates were recorded and data were descriptively analysed.

**Results:**

Thirty-one patients were recruited across three sites. Eighteen staff and 18 patients took part in interviews, and 19 patients returned questionnaire sets. All four progression to trial criteria were met. We identified barriers to patient engagement with the intervention in hospital, which were compounded by patients’ focus on returning home. Some patients described not engaging in discussions with staff about medicines and lacking motivation to do so because they were preoccupied with returning home. Some patients were unable or unwilling to attend a community pharmacy in person for a medicines review. Roles and responsibilities for delivering the MaTI were different in the three sites, and staff reported variations in time spent on MaTI activities. Staff reported some work pressures and staff absences that limited the time they could spend talking to patients about their medicines. Clinical teams reported that recording a target dose for heart failure medicines in patient-held documentation was difficult as they did not always know the ideal or tolerable dose. The majority of patients reported receiving the patient-held documentation. More than two-thirds reported being offered a MUR by their community pharmacists.

**Conclusions:**

Delivery of the Medicines at Transitions Intervention (MaTI) was feasible at all three sites, and progression to trial criteria were met. Refinements were found to be necessary to overcome identified barriers and strengthen delivery of all steps of the intervention. Necessary changes to the MaTI were identified along with amendments to the implementation plan for the subsequent trial. Future implementation needs to take into account the complexity of medicines management and adaptation to local context.

## Key messages regarding feasibility

- It was uncertain whether the Medicines at Transitions Intervention could be initiated by a ward team in an acute hospital setting and continued by a community pharmacist for heart failure patients moving across a care transition. It was uncertain whether patients could be recruited at the required rate for a cluster randomised controlled trial to be conducted.
- It was possible to deliver the intervention across the care transition. The intervention was found to be feasible and acceptable in three acute trusts and in the community, although some problems occurred when patients were discharged outside core hours. Some information required to deliver some aspects of the intervention, such as target medication doses, was not available to staff.
- Changes were recommended to enhance effective communication between cardiology ward and hospital pharmacy staff and provide guidance on discharges occurring at night and weekends, and the requirement to complete dose titration information within the MaTI was removed.

## Introduction

Heart failure affects 26 million people globally, and poor outcomes for patients who have been hospitalised as a result of their heart failure have not improved in line with advances made in evidence-based treatment [1]. There is also a significant economic burden associated with being hospitalised and readmitted as a result of the condition [2]. Heart failure is a condition for which there is strong evidence for the therapeutic benefit of combinations of medicines at titrated doses [3]. Thus, the optimal management of medicines is crucial to prevent readmission to hospital; it can also improve quality of life and increase the survival rate [4]. However, the management of medicines is a complex process that involves patients, their carers, and (potentially) multiple healthcare professionals working in different organisations [5, 6]. Ensuring optimal clinical outcomes with minimal harm requires well-calibrated cross-organisational processes with staff and patients responding and adapting to changes such as medicines changes made by different providers, patient response to treatment, patient capabilities to manage medicines routines, and patient co-morbidities [7]. Errors associated with medicines management can be significant [8, 9], and the challenges for preventing these are greater when healthcare providers handover care, for example, on discharge from hospital when medicines changes need to be communicated with the patient, their carers, and the patient’s family doctor (General Practitioner; GP) [10]. In the UK, other healthcare professionals who are part of the medicines management system are not routinely made aware of these medicines changes by hospitals; this includes community pharmacists who dispense patients’ medicines and support their use. Thus, it has been argued that the implementation of evidence-based medication therapy could be improved [11, 12].

We have found that there are opportunities to support patients in playing a more informed and proactive role in the management of their medicines [13]. and for healthcare professionals, such as community pharmacists, to be more meaningfully integrated into the medicines management system [14]. We have also identified that despite multiple opportunities for patient safety incidents to occur, staff and patients are able to build resilience into this system through temporary or permanent fixes [7]. Successful interventions to improve medicines management when care is transferred include enhanced patient education and better healthcare professional communication [15]. However, whole patient pathway approaches are lacking and there are few interventions that have been co-designed with their patient and staff users.

The aim of this study was to assess the feasibility of implementing a complex intervention (the Medicines at Transitions Intervention (MaTI)) co-designed by patients and healthcare staff as part of the programme. Intervention feasibility was assessed through a set of specified progression to trial criteria. The purpose of the intervention was to optimise medicines management across the gaps between secondary and primary care when hospitals handover care. In particular, the work reported here is intended to evaluate the acceptability and deliverability of the intervention by assessing whether the target patient population could be recruited and whether data collection tools were fit for purpose. Following Medical Research Council (MRC) guidance [16], the study was conducted to enable further refinement of the MaTI and identify potential implementation problems in advance of a proposed cluster randomised control trial (cRCT) focussed on the reduction of readmission and all-cause mortality measured through routine data sources. An additional aim was to enhance the implementation of the MaTI in the clinical context for heart failure medicines management.

Therefore, the objectives of this study were to:

1. Achieve the specified progression criteria to proceed to the trial;
2. Assess if the intervention components were feasible to deliver and acceptable to staff and patients and if the trial data could be collected;
3. Determine whether amendment or refinement of any intervention components would be needed in order to enhance deliverability and acceptability.

## Methods

We adopted the Consolidated Framework for Implementation Research (CFIR) [17] as our overall framework for the development and evaluation of the intervention. A favourable ethical opinion was provided by a UK National Health Service Research Ethics Committee (17/YH/0128). Although this was a non-randomised feasibility study, where applicable, the principles of the CONSORT extension for randomised pilot and feasibility trials have been adhered to in reporting [18]. Objective 1 was assessed through numbers recruited to the study at each site and through questionnaires returned by patients; objective 2 was assessed through site checklists and qualitative semi-structured interviews with staff and patients and questionnaires; objective 3 was assessed through qualitative semi-structured interviews with staff and patients.

### About the intervention

We followed the MRC Framework for intervention development [16] and conducted a multi-site Experience-Based Co-Design (EBCD) [19] process with heart failure patients who had recently been discharged from hospital, together with their carers and their healthcare teams [20]. The prototype intervention was mapped onto behaviour change techniques and refined based on feedback from a multi-stakeholder panel, including commissioners, healthcare professionals, and patients.

**Fig. 1:**
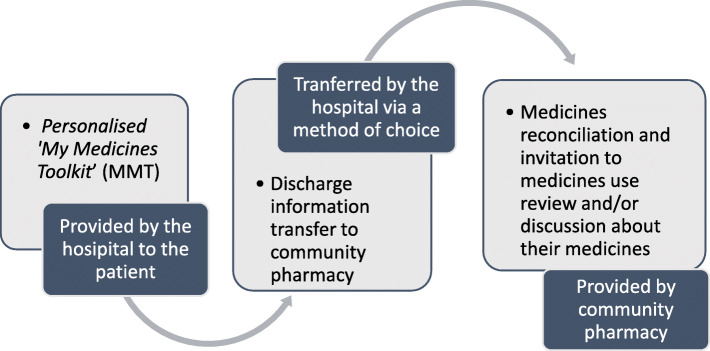
The Medicines at Transitions Intervention

Briefly, the resultant MaTI intervention consisted of multiple, complementary components designed to optimise medicines management across a common ‘gap’ in care when responsibility for patients is transferred between clinicians in different organisations [21]. It is described in Fig. 1 and comprised:

- A patient-held information resource ‘*My Medicines Toolkit*’ (MMT) for patients comprising information about their heart failure medicines, their healthcare team, and symptoms, plus a checklist for them to complete detailing events that should have happened in hospital, at discharge, and after discharge;
- Transfer of discharge information to community pharmacy to encourage and facilitate medicines reconciliation [22];
- An invitation to attend a post-discharge medicines use review (MUR) and/or discussion about their medicines with the community pharmacist [23].

Intervention checklists were implemented at each site to track which components of the intervention were delivered, and a follow-up checklist was sent to community pharmacies after patients were discharged to track post-discharge intervention delivery. The hospital and community pharmacy checklists were returned to the study team for analysis.

### Progression to trial criteria

In advance, the study’s independent Programme Steering Committee (comprising academics, clinicians, and a patient representative) and the funder agreed four criteria for the feasibility study to progress to trial, with associated ‘Red’, ‘Amber’, and ‘Green’ (RAG) criteria that were set to measure and assess performance (Table 1). The RAG approach was chosen to align with methods established for trials using ‘stop’, ‘change’, and ‘go’ progression criteria [24].

**Table 1:** Feasibility assessed through progression to trial criteria and their RAG boundaries: if Green, then progression is feasible; if Amber, then review of the intervention procedure would be necessary; if Red, then the component of the study would not be deemed deliverable during the cRCT

Criterion	RAG
1. Recruitment of a minimum of one patient per week per site (30 participants in 10 weeks across three sites). Assessed at 10 weeks after start of feasibility recruitment in each site	*Green*: 8–10 patients *Amber*: 5–7 patients *Red*: 5 patients
2. Patient reported receipt of My Medicines Toolkit (MMT)/Site checklist confirms patients received MMT	*Green*: Evidence that patients received the MMT in all three trusts *Amber*: Evidence that patients received the MMT in two of three trusts *Red*: MMT only delivered in one trust
3. Transfer of discharge advice and medicines letter to community pharmacies in all three sites—confirmed either by site checklist OR community pharmacy follow-up	*Green*: Evidence that all three trusts able to communicate discharge advice letter to community pharmacy *Amber*: Evidence that two trusts able to transfer this information *Red*: Only evidence that one trust is able to transfer information
4. Patients report/community pharmacy report the offer of a community pharmacy medicines review or medicines discussion OR community pharmacy reports completion of medicines reconciliation	*Green*: Evidence that community pharmacies in all three areas able to offer review or perform medicines reconciliation *Amber*: Evidence that community pharmacies in two areas able to offer these services *Red*: Evidence that it has only been possible for community pharmacies in one area to offer these services

### Study setting and patient recruitment

We tested the feasibility of the MaTI in three healthcare areas in the North of England between July and October 2017. From the cardiology wards of each of the three NHS Acute Trusts (sites), we recruited 10 patients (aged ≥ 18 years) hospitalised with evidence of at least moderate left ventricular systolic function (left ventricular ejection fraction < 45%) confirmed within the last 5 years, and the capacity to provide informed consent. The sample size was calculated from the first feasibility criterion of recruiting one patient per week per site over the course of the study. This target was important to establish the deliverability of the subsequent clinical trial. In addition, the qualitative components of the study required the perspectives of 20 patients; we predicted that, with dropout, 30 recruited patients would yield that number of interviews. Patients were assessed for eligibility and approached on the ward by a research nurse who explained the study and provided a patient information leaflet, and if the patient agreed to participate they gave written informed consent.

### Delivering the intervention

Staff were trained to deliver the intervention by a member of the research team. As staff roles and responsibilities vary on cardiology wards between sites, we did not specify which staff should deliver which components of the intervention: this was decided by sites based on local staffing. Following recruitment and before their discharge from hospital, patients were introduced to the personalised MMT by a member of their clinical team. Staff were also expected to complete a discharge medicines list in the patient's MMT. Patients were asked to identify their usual community pharmacy or nominate a pharmacy if they did not usually use one. At discharge, the discharge advice letter (including the medicines list) was transferred to the community pharmacy, either by fax or electronically depending on the hospital’s usual method, along with a written request that the community pharmacist will reconcile the patient’s medicines and invite the patient to a MUR or discussion about their medicines.

### Patient follow-up interviews and surveys

At 1 month following discharge from hospital, all patients were posted a set of three questionnaires for self-completion: quality-of-life (EQ 5D 3L) [25], healthcare utilisation (to capture economic data), and a patient experience survey (PES). The PES was developed based on the findings of a multi-site heart failure medicines management pathway analysis conducted as part of intervention development work, and its purpose was to explore patient experiences of receiving the intervention and with their medicines [7]. The questions in the survey explored what patients were told about their medicines at the time of their discharge from hospital, their use of the My Medicines Toolkit (MMT), whether they were at least offered a MUR or medicines discussion by their community pharmacist after they had left hospital, and if they actually had a medicines use review. The EQ 5D 3L and healthcare utilisation questionnaire were implemented in this feasibility study to determine if they could be completed and returned by patients, and their responses are not reported in this paper. In a purposive sample, 20 patients were invited to participate in a semi-structured interview about their experiences of the MaTI and how their medicines were managed after they left hospital. An interview schedule was developed exploring their experiences, and, following gaining written consent, interviews were undertaken at patients’ homes. A detailed account of the methods, analysis and results of the qualitative stage will be reported in a subsequent paper. Here, we combine headlines of the qualitative results as a component of the whole set of study data.

### Staff follow-up interviews

Staff from hospital and primary care settings involved in patients’ care were invited to participate in a qualitative face-to-face interview and were provided with a study information leaflet. Staff approached for interviews included hospital and community pharmacists, specialist heart failure nurses, ward nurses, and hospital doctors. If they agreed to participate, they signed a consent form. An interview schedule was constructed to explore staff perceptions of the acceptability and deliverability of the intervention and their views about the medicines management pathway. As above, these data will be reported in full in a subsequent paper.

### Data analysis

Patient and staff interviews were audio recorded, transcribed verbatim, and analysed using Framework Analysis—data were coded and a framework was constructed that contained themes describing patients’ medicines experiences [26]. Questionnaire completion rates were recorded, and data were descriptively analysed using SPSS v.21 [27].

## Results

Overall, 31 patients were recruited across the three sites. At 1 month following discharge from hospital, three patients had been readmitted to hospital, one patient had died and six could not be contacted or did not wish to continue. Table 2 shows the number of patients recruited from each site and those retained. Table 3 shows the characteristics of recruited patients. Nine patients were women, 27 were White British, and four were British Asian, and the mean age was 70 (range 38–93).

**Table 2:** The number of patients recruited and retained at each site

	Site 1	Site 2	Site 3	Total
Number of patients recruited	10	11	10	31
Number of patients readmitted	2	0	1	3
Number of patients died	0	1	0	1
Number of patients who could not be contacted or did not wish to continue	1	4	1	6
Number of patients retained through to follow-up	7	6	8	21

**Table 3:** The characteristics of recruited patients

	Site 1	Site 2	Site 3	Totals
Women	2	3	4	9
Male	8	8	6	22
White British	10	9	8	27
British Asian	0	2	2	4
Mean age (median, range), years	63 (61, 38–84)	78 (80, 67–93)	69 (65, 64–81)	70 (65, 38–93)
Total number of patients	10	11	10	31

### Study performance against progression criteria

All progression to trial criteria were met during the feasibility study, and the more detailed evidence from patient questionnaires, hospital site checklists, and community pharmacy questionnaires is detailed in Table 4.

**Table 4:** Performance against progression criteria

Criterion	Evidence
1 Recruitment	All three sites recruited at least ten patients. Two sites did this within 10 weeks and one site required two additional working days.
2 Patient receipt of MMT	Implementation of the MMT was delivered in all three sites. Study checklists were returned from site 1 (ten), site 2 (eight), and site 3 (ten) and indicated that all patients received the toolkit. These data were corroborated by patient reports from 19 returned questionnaires from site 1 (eight), site 2 (three), and site 3 (eight) indicating that 17/19 patients received the MMT. In site 1, one patient could not remember receiving it; in site 3, one patient reported that they did not receive it (however the site checklist records that they did receive it and the patient answered other questionnaire items suggesting that they did in fact receive the MMT).
3 Transfer of discharge information	All three sites were able to adapt systems so that hospital pharmacists transferred this information. Checklists from sites 1 and 3 indicate that the information was transferred for all patients. The eight study checklists returned from site 2 indicate that information was transferred for four patients. This was because the hospital pharmacist mistakenly thought that this should not be done if they had not themselves seen the patient before discharge. These data were corroborated by community pharmacy data returns: in site 1, eight reported receiving the patient’s discharge information; 4 reported doing so in site 2; and eight did so in site 3.
4. Offer of a community pharmacy medicines review or medicines discussion OR medicines reconciliation	Community pharmacies in all three areas reported acting on hospital information and reconciling medicines or where appropriate inviting the patient for a Medicines Use Review (MUR) or discussion about their medicines. Community pharmacy data returns were received from nine pharmacies in site 1, eight in site 2, and eight in site 3: In site 1, medicines reconciliation was performed for eight patients; all eight were offered a MUR/discussion. In site 2, medicines reconciliation was performed for four patients, two of whom were offered MURs or medicines discussions. In site 3, medicines reconciliation was performed for eight patients, seven of whom were offered a MUR or medicines discussion. Of the 19 patient survey respondents, 13 reported being offered a review: six from site 1, one from site 2, and six from site 3.

### Patient and staff experiences of the intervention

Interviews were conducted with 18 patients: seven from site 1, three from site 2, and eight from site 3; two of those interviews were held with a family member who helped translate. Additionally, interviews were conducted with 18 healthcare professionals: three hospital heart failure specialist nurses, three consultant cardiologists, three cardiology ward pharmacists, six community pharmacists, and three community heart failure nurses. The in-depth findings of the qualitative data analysis will be reported in a separate paper. In summary, we identified barriers to patient engagement with the intervention in hospital that were compounded by patients’ focus on returning home. Most patients remembered receiving the MMT and the member of staff who had introduced it to them. Some patients described not having engaged in discussions with staff about medicines and lacking motivation to do so due to preoccupation with returning home. Some patients were unable or unwilling to attend a community pharmacy in person for a MUR and none reported being offered the option of a telephone MUR.

Roles and responsibilities for delivering the MaTI varied in the three sites, and staff reported variations in time spent on MaTI activities. They reported some work pressures and staff absences that limited the time they could spend talking to patients about their medicines. Specialist heart failure nurses felt that going through the toolkit would be more difficult for non-specialist nurses as they would not have the required specialist knowledge. Clinical teams reported that including a target dose in the MTT for heart failure medicines was not straightforward as they did not always know what that was or whether patients could tolerate it. Staff also reported that good internal communication—particularly between ward and pharmacy staff—was required for successful delivery of the MaTI, and their accounts showed considerable variation across the three sites. Although there was no pre-existing routine communication with community pharmacy, hospital staff were able to transfer information to patients’ community pharmacies. IT systems such as PharmOutcomes® facilitated this transfer where available. However, other forms of information transfer such as paper-based meant that information was sometimes delayed or did not arrive at the community pharmacy. If cardiology pharmacy staff did not see patients prior to discharge, for example, if the discharge took place in the evening or at weekends, information was not always transferred. Community pharmacists described barriers to inviting patients or patients attending a review or discussion about their medicines, such as patients not being well enough to travel to the pharmacy. However, having discharge information had enabled community pharmacists to reconcile medicines and conduct MURs more meaningfully.

### Patient experience survey responses

In total, 19 patients (of 21 available) responded, indicating that it was possible to collect survey data from patients following discharge from hospital and that patients were able to answer most questions on the PES: four questions required amendments to aid interpretation. Responses to questions related to deliverability and feasibility of the intervention are summarised in Table 5. The majority (17) of respondents reported receiving the MMT, six of whom reported not using it. More than two-thirds (13) reported being offered a MUR by their community pharmacist; however, fewer patients than pharmacists reported that a MUR had taken place.

**Table 5:** Responses to the patient experience survey (*n* = 19)

Question	***N***
Patient given a copy of MMT	17
Patient used MMT:	
No	6
Between 1 and 5 times	6
Between 6 and 10 times	5
More than 15 times	1
Patient offered a community pharmacy medicines use review	13

## Discussion

We found that it was feasible to deliver the MaTI in three healthcare areas including hospitals and community pharmacies and that it was possible to recruit from the target population for the intervention. Study checklists detailing the intervention steps were returned from all three sites, which indicated that patients received the toolkit. Hospitals were able to adapt systems so that discharge information was transferred to community pharmacy. Community pharmacies were able to act on hospital information by reconciling medicines and where appropriate inviting the patient for a MUR or discussion about their medicines. The majority of patients who responded to the patient experience survey reported that they had been offered a review by the community pharmacist. We demonstrated during the feasibility study that there was a patient population for our planned subsequent cRCT. Our criterion of 10 patients recruited in 10 weeks was met by all sites.

There are clear implications from our study for implementing this type of complex intervention in multiple healthcare settings. First, implementation needs to take into account the complexity of medicines management and adaptation to local context needs to be considered [28]. Current thinking about complex intervention design and implementation recommends a fresh focus on the local environment in planning flexible implementation and context-specific facilitation, and our study supports this view [29]. Secondly, and closely linked, training needs to make clear how to proceed if intervention steps are missed due to the complexity of care. Assumptions about the dependency of intervention components also impacted on delivery: some staff thought that missing one step in the intervention meant that they should not deliver subsequent intervention steps. This resulted in intervention stages being left incomplete, for example, if they did not complete the patient’s fold-out medicines chart in the MMT, they then did not transfer information to community pharmacy. Learning about this misconception allowed us to adapt the intervention training package accordingly. Additionally, we found in some cases that when components of the intervention were completed they were not always recorded on the checklists.

There were also important implications for scale-up to a trial; for example, we developed information for the trial implementation guide that encourages active monitoring of patient checklists to ensure each intervention step is taken for every patient. We found that ward staff do not routinely find out which community pharmacy the patient uses, so we developed guidance to help staff to do this and our on-site training includes a session on helping patients nominate a community pharmacy. We also introduced a step where the staff member who transfers discharge information contacts the community pharmacies by telephone to check that the information has been received. Finally, we introduced a script for staff to use that can help guide them through the medicines discussion with patients.

In addition, staff from different teams needed to be available to deliver parts of the intervention to patients before discharge including out of hours and at weekends. However, some staff such as pharmacy staff and specialist heart failure nurses were not always available to see patients in a timely way. Moreover, the focus on returning home for patients meant that they were not always receptive to receiving medicines information at this point in their care. Hospitals also had differing systems for communication with primary healthcare teams. The subsequent intervention implementation package developed for the cRCT by our team ensured that staff could be prepared for these situations and a site coordinator role was developed for implementation sites to coordinate intervention delivery locally.

We found that the roles and responsibilities allocated to different healthcare professions on cardiology wards varied. For example, discussing medicines with patients may be undertaken by ward staff, hospital-based heart failure nurses. and pharmacy staff. This confirmed our intention that we should not specify which staff members needed to undertake which intervention steps, and in our trial intervention sites, the clinical team decided how to allocate the intervention steps to different staff types based on local staff mix and practice. Good team working and communication are essential components of safe care [30], and we found that effective communication was necessary between nursing and pharmacy staff to deliver the MaTI intervention. This included communication about the timing of discharge so that the pre-discharge intervention components could be delivered, for example, completion of the medicines chart which was part of the MMT and the medicines that the patient would be taking home. This informed the design of our intervention training package and implementation materials.

The hospitals did not routinely transfer discharge information to community pharmacy prior to this study, despite the role that pharmacists can play in identifying post-discharge medicines problems [31, 32]. We found that transferring information about heart failure medicines was possible in all three sites, but was not always achieved in a timely fashion, and community pharmacy did not always receive this information. Hospitals had different systems to transfer information, including post and fax; one site used PharmOutomes®, a web-based system that allows messaging between sites. Whilst use of this type of technology is gradually increasing, our intervention needed to be implementable in sites still using paper or fax, so in the final intervention we added a step for the hospital to contact the community pharmacist to verify that the information had been received.

Whilst heart failure medicines should be titrated to evidence-based target doses [4], we found that staff did not always agree with providing a target dose for ACE inhibitors, angiotensin receptor blockers, and/or beta-blockers in the patient’s fold-out medicines chart. Some staff told us that they did not always know what the target dose was and if they did whether the patient would tolerate that dose. Consequently, we removed the target dose from the subsequent intervention; however, the MMT still indicated that some treatment doses were intended to be gradually increased once they were back at home.

The strengths of this study lie in its multi-site delivery, which included different geographical areas. It also comprised a robust assessment of the feasibility of a complex intervention across a care transition against challenging progression criteria using the infrastructure that would be in place during a clinical trial. Therefore, we consider the methods used would be transferable to other feasibility studies. We also tested different methods of transferring discharge information to community pharmacy.

There are also limitations to take into account. For example, we knew prior to the study that there would be a larger number of heart failure patients who met the clinical inclusion criteria for the study, but were treated on non-cardiology wards (for example Care of Older People wards), and this was the case in each of the feasibility study sites. The National Institute of Health and Care Excellence (NICE) heart failure guidance stipulates that patients should be treated by specialist cardiology teams, and this patient population may have been visited by heart failure specialist nurses during their in-patient stays if nurses were available and identified as needing this specialist care. However, our study was limited to patients on cardiology wards because it is a ward-level team-based intervention. Furthermore, we knew it would be tested during a clinical trial in sites that may not have heart failure outreach service for patients on non-cardiology wards.

## Conclusions

Delivery of the Medicines at Transitions Intervention (MaTI) was found to be feasible and acceptable at three test sites, and progression to trial criteria were met. Areas for refining hospital implementation were establishing effective communication between cardiology ward and pharmacy staff, providing guidance on discharges occurring at night and weekends, and developing an intervention co-ordination role. In primary care, community pharmacists reported making use of discharge medicines information to reconcile patients’ medicines but some patients were unable to attend the pharmacy for a MUR. Refinements were made to strengthen delivery of all steps of the intervention and to the implementation plan for the trial.

## Data Availability

The datasets generated and analysed during the current study are not publicly available due to consent restrictions.
